# Identification of Novel Mutations in *HEXA* Gene in Children Affected with Tay Sachs Disease from India

**DOI:** 10.1371/journal.pone.0039122

**Published:** 2012-06-18

**Authors:** Mehul Mistri, Parag M. Tamhankar, Frenny Sheth, Daksha Sanghavi, Pratima Kondurkar, Swapnil Patil, Susan Idicula-Thomas, Sarita Gupta, Jayesh Sheth

**Affiliations:** 1 FRIGE Institute of Human Genetics, Ahmedabad, Gujarat, India; 2 ICMR Genetic Research Center, National Institute for Research in Reproductive Health, Mumbai, Maharashtra, India; 3 Biomedical Informatics Center, National Institute for Research in Reproductive Health, Mumbai, Maharashtra, India; 4 Department of Biochemistry, M.S. University, Vadodara, Gujarat, India; Charité Universitätsmedizin Berlin, NeuroCure Clinical Research Center, Germany

## Abstract

Tay Sachs disease (TSD) is a neurodegenerative disorder due to β-hexosaminidase A deficiency caused by mutations in the *HEXA* gene. The mutations leading to Tay Sachs disease in India are yet unknown. We aimed to determine mutations leading to TSD in India by complete sequencing of the *HEXA* gene. The clinical inclusion criteria included neuroregression, seizures, exaggerated startle reflex, macrocephaly, cherry red spot on fundus examination and spasticity. Neuroimaging criteria included thalamic hyperdensities on CT scan/T1W images of MRI of the brain. Biochemical criteria included deficiency of hexosaminidase A (less than 2% of total hexosaminidase activity for infantile patients). Total leukocyte hexosaminidase activity was assayed by 4-methylumbelliferyl-N-acetyl-β-D-glucosamine lysis and hexosaminidase A activity was assayed by heat inactivation method and 4-methylumbelliferyl-N-acetyl-β-D-glucosamine-6-sulphate lysis method. The exons and exon-intron boundaries of the *HEXA* gene were bidirectionally sequenced using an automated sequencer. Mutations were confirmed in parents and looked up in public databases. *In silico* analysis for mutations was carried out using SIFT, Polyphen2, MutationT@ster and Accelrys Discovery Studio softwares. Fifteen families were included in the study. We identified six novel missense mutations, c.340 G>A (p.E114K), c.964 G>A (p.D322N), c.964 G>T (p.D322Y), c.1178C>G (p.R393P) and c.1385A>T (p.E462V), c.1432 G>A (p.G478R) and two previously reported mutations. c.1277_1278insTATC and c.508C>T (p.R170W). The mutation p.E462V was found in six unrelated families from Gujarat indicating a founder effect. A previously known splice site mutation c.805+1 G>C and another intronic mutation c.672+30 T>G of unknown significance were also identified. Mutations could not be identified in one family. We conclude that TSD patients from Gujarat should be screened for the common mutation p.E462V.

## Introduction

Tay Sachs disease (TSD) (MIM# 272800) is an autosomal recessive neurodegenerative disorder due to β-hexosaminidase A deficiency caused by mutation in the *HEXA* gene (MIM* 606869) encoding the α subunit of hexosaminidase A, a lysosomal enzyme composed of α and β polypeptides [1]. The clinical picture ranges from the acute infantile form rapidly leading to death to progressive later onset form compatible with a longer survival. Clinical features include neuroregression, generalized hypotonia, exaggerated startle response and cherry-red spot seen on fundus examination. Affected patients have deficient enzyme activity of Hexoasaminidase A (Hex A) in leukocytes or plasma [2]. The human *HEXA* gene is located on chromosome 15 q23-q24 with 14 exons. Nearly 130 mutations have been reported so far in the *HEXA* gene to cause TSD and its variants, including single base substitutions, small deletion, duplications and insertions splicing alterations, complex gene rearrangement and partial large duplications (http://www.hgmd.cf.ac.uk/). Most of these mutations are private mutations and have been present in a single or few families. Only few mutations have been commonly found in particular ethnicities or geographically isolated populations. In the Ashkenazi Jews, 94 to 98% patients are caused by one of the three common mutations c.1277_1278insTATC, c.1421+1 G>C and c.805 G>A (p.G269S) [3]–[5]. Among the non-Ashkenazi TSD patients the mutations pattern is completely different. A 7.6 kb deletion which includes the entire exon 1 and parts of the flanking sequence, is the major mutation causing TSD in the French Canadian population [4]. The mutation c.571–1 G>T accounts for 80% of mutant alleles among Japanese patients with TSD [1]. The c.1277_1278insTATC and the c.805 G>A(p.G269S) mutations are also commonly found in non-Ashkenazi Jewish populations, along with an intron 9 splice site mutation (c.1073+1 G>A) and the 7.6 kb French Canadian deletion. About 35% of non-Jewish individuals carry one of the two pseudodeficiency alleles; c.739C>T (p.R247W) and c.745C>T (p.R249W), which are not associated with neurological manifestations, since their presence causes the reduction of Hex A activity only towards the artificial substrate but not to the natural GM2 ganglioside [6]. The mutations responsible for TSD in Indian patients are hitherto unpublished.

## Results

Fifteen families were included in the study. Consanguinity in parents was present in 4/15 (26.7%) families. The mean age at presentation was 16.6 months (+/−5.4). All patients had seizures, neuroregression, exaggerated startle reflex, cherry red spot on fundoscopy, axial hypotonia, increased peripheral limb tone and brisk deep tendon reflexes. None of the patients had hepato-splenomegaly. Neuroimaging in form of computed tomography (CT scan) or magnetic resonance imaging (MRI) of the brain of the proband was available in 10/13 patients and showed characteristic findings of putaminal hyperintensity and thalamic hypointensity in CT scan or T2 weighted images of MRI of the brain. There were no white matter abnormalities. Significant deficiency of Hex A activity was observed in the leukocytes of all thirteen patients and only carrier detection (% Hex A) could be performed in two families where the proband was not alive (Table 1).

**Table 1:** Clinical, biochemical and molecular details of the Indian patients with Tay Sachs disease.

S no	Age at diagnosis	Native State	Consan- guinity	Hex-A activity (MUGS) (nmol/hr/mg) = (x)	Total Hex activity (MUG) = (y)	Hex A % = (x/y) X 100	Genotypes (nucleotide level)	Genotypes (protein level)
1	11 months	Gujarat	No	4.5	ND	-	c.1385A>T/c.1385A>T	p.E462V/p.E462V
2	1.5 Yrs	Gujarat	No	4.8	1827.6	0.2	c.1385A>T/c.1385A>T	p.E462V/p.E462V
3	1 Yrs	Gujarat	No	5.9	2208.9	0.26	c.1385A>T/c.1385A>T	p.E462V/p.E462V
4	1.5 Yrs	Gujarat	No	5.1	2009	0.25	c.1385A>T/c.1385A>T	p.E462V/p.E462V
5	1.5 Yrs	Gujarat	No	5.8	2288	0.25	c.1385A>T/c.1385A>T	p.E462V/p.E462V
6	11 months	Gujarat	No	0.0 Father -60% (HLC) Mother –53% (HLC)	2131.0 731.7 528.3	0.0 - -	c.1385A>T/c.1385A>T	p.E462V/p.E462V
7	2.5 Yrs	Uttar Pradesh	No	4.08	1453.0	0.28	c.964 G>A/c.964 G>A	p.D322N/p.D322N
8*	15 months	Uttar Pradesh	No	0.0	716.2	0.0	Father and mother - [c.964 G>T/−]	p.D322Y/−
9	10 months	Maharashtra	No	23.8	1840.2	1.29	c. 340 G>A/c.340 G>A	p.E114K/p.E114K
10	1 Yrs	Maharashtra	No	7.2	2100	0.3	c.672+30T>G/c.1432 G>A	Undetermined/p.G478R
11	18 months	Tamil Nadu	Yes	17.2	2054.5	0.83	c.1178 G>C/c.1178 G>C	p.R393P/p.R393P
12*	1 Yrs	Andhra Pradesh	Yes	Father- 59.3% (HLC) Mother - 59.1% (HLC)	ND	-	Father -[c.805+1 G>C/−] Mother -[c.805+1 G>C/−]	-
13	1.5 Yrs	Iraq	Yes	18.4	1776.9	1.03	c.508C>T/c.508C>T	p.R170W/p.R170W
14	1 Yrs	Karnataka	Yes	1.5	1660	0.09	Not found	-
15	16 months	Gujarat	No	2.1	ND	_-	Father and mother - [c.1277_1278insTATC/−]	-

* DNA of index case is not available, HLC-heat labile activity in carrier parents, ND – not done.

Normal total hexosaminidase values using MUG substrate in our controls –703 to 1785 nmol/hr/mg protein, normal hexosaminidase A levels – (62 to 77%); normal MUGS activity 80 to 390 nmol/hr/mg.

**Figure 1:**
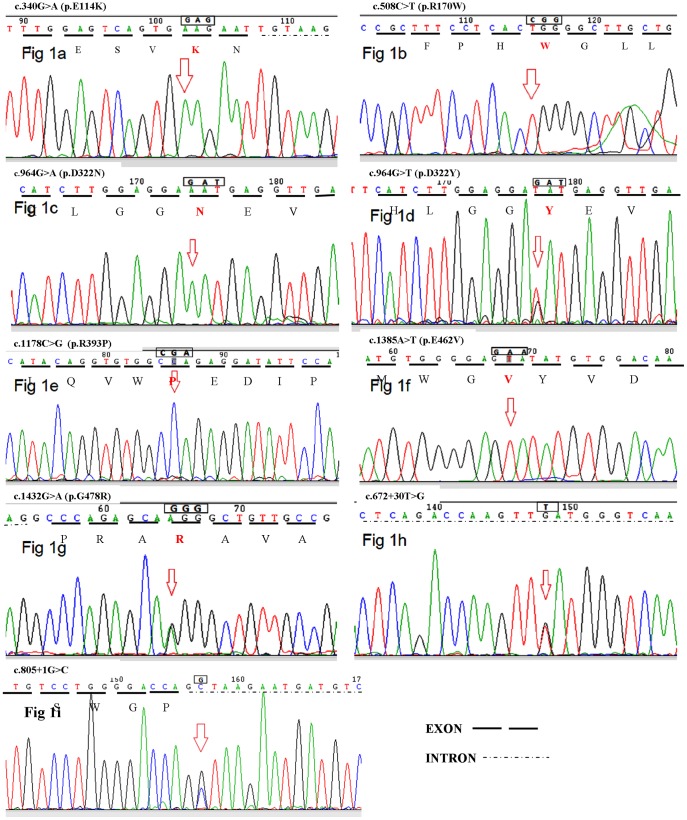
(a) – (e): Sequence Chromatogram of mutations Fig. 1a: c. 340 G>A (p.E114K)(homozygous); Fig. 1b: c.508C>T (p.R170W)(homozygous); Fig. 1c: c.964 G>A (p.D322N)(homozygous); Fig. 1d: c.964 G>T (p.D322Y)(heterozygous); Fig. 1e: c.1178 G>C (p.R393P) (homozygous); Fig. 1f: c.1385A>T (p.E462V) (homozygous); Fig. 1g: c.1432 G>A (p.G478R)(heterozygous); Fig. 1h: c.672+30T>G (heterozygous); Fig. 1e: c.805+1 G>C.

The DNA of fourteen such affected TSD families did not have the common mutations c.1277_1278insTATC, c.1421+1 G>C, c.805 G>A (p.G269S), 7.6 kb deletion or the two pseudodeficiency mutations c.739C>T (p.R247W) and c.745C>T (p.R249W). The common mutation c.1277_1278insTATC was detected by screening in one family and was confirmed by sequencing. Complete sequencing analysis revealed nine different mutations in thirteen families and could not identify any mutation in one family. We identified six novel deleterious missense mutations, c.340 G>A, c.964 G>A, c.964 G>T, c.1178C>G and c.1385A>T, c.1432 G>A, that resulted in amino acid changes p.E114K, p.D322N, p.D322Y, p.R393P, p.E462V, p.G478R [refer to Figure 1(a)–(d)]. We also identified known pathogenic mutations c.508C>T (p.R170W), c.805+1 G>C and another intronic variant c.672+30T>G of undetermined significance (refer to Table 1). The novel mutations were not found in 100 control individuals. The missense mutations were not found in the 1000 Genome Project. The intronic variant c.672+30T>G (rs117160567) was found in 1000 Genome Project (Minor Allele Frequency/Minor Allele Count: C = 0.09/20). The mutation c.1385A>T (p.E462V) was found in the homozygous state in six acute infantile TSD patients belonging to unrelated families with common ethnicity (from Gujarat) indicating a founder effect. Haplotype analysis was performed by sequencing the introns 1, 5, 12, 13 and a 417 basepair region 3′ to HEXA gene which contained several polymorphic markers, in all TSD patients and 30 ethnic controls. Table 2 reveals the haplotypes unique to the founder mutation and not found in other patients and 30 ethnic controls. The founder mutation was screened in 500 individuals using ARMS–PCR (amplification refractory mutation system- polymerase chain reaction) and two individuals were found to be carrier for this mutation leading to an allele frequency of 1/500. The ARMS method used the primers.

**Table 2:** Haplotype analysis of founder mutation p.E462V in Gujarati patients with Tay Sachs disease.

3′ to HEXA gene(a)	Al	Intron 13 (b)	Al	Intron 12 (c)	Al	Intron 5 (d)	Al	Intron 1 (e)	Al
rs35949555	T	rs12904378	C	rs74020947	G	rs191809305	A	rs191094610	A
rs11629508	C	rs113387077	A	rs57733983	T	rs188570040	T	rs188410016	C
rs76075374	C	rs2912217	G	rs189856670	A	rs60920713	C	rs186683578	A
rs60288568	G	rs190224431	G	rs140288703	G	rs59427837	C	rs184065715	T
rs112626309	G	rs145393752	T	rs185764548	C	rs12910617	C	rs78629973	C
rs3087652	T	rs112806142	G	rs111680766	A	rs12593333	A	rs4470105	C
		rs890313	G	rs147324677	C	rs12592727	T	rs78278321	G
		rs111827252	C	rs113941121	A	rs4776594	G	rs76530364	T
		rs75015614	T	rs34085965	A			rs75756977	C
		rs62022857	C	rs2303448	C			rs75981720	C
		rs118002327	C					rs76941148	C
		rs2912218	T					rs80039124	T
		rs113665670	CTCT					rs78970750	A
		rs149948017	T					rs76950885	C
		rs4777502	C					rs74738827	A
		rs147502219	T					rs80238386	G
		rs140091006	G					rs77154656	C
		rs145012038	C					rs78097627	C
		rs149092488	G					rs77511366	A
								rs74325922	G
								rs74787391	C

(Al  =  allele).

Region (a)  = 417 bp region 3′ to HEXA gene (chromosome 15 plus strand: 72635626 to 72636042); region (b)  = 405 bp region in intron 13 of HEXA gene (chromosome 15 plus strand: 72637019 to 72637423; and 567 bp region in intron 13 of HEXA gene (chromosome 15 plus strand: 72637428 to 72637994; region (c)  = 489 bp region in intron 12 of HEXA gene (chromosome 15 plus strand:72638306 to 72638794); region (d)  = 424 bp region in intron 5 of HEXA gene (chromosome 15 plus strand: 72639456 to 72639879); region (e)  = 405 bp region in intron 1 of HEXA gene (chromosome 15 plus strand: 72656546 to 72656950 and 461 bp region in intron 1 of HEXA gene chromosome 15 plus strand: 72659130 to 72659590). The chromosome.

coordinates are as per Human Genome Assembly Feb 2009 (GRCh37/hg19).

5′-CCAGGTTTGTGTTGTCCACATATT-3′ (mutation specific primer) and 5′-AGTTACCCCACCATCACCAGACTG-3′ (common forward primer) in a PCR reaction to show presence of p.E462V allele (123 base pair PCR product) and the primers 5′-CCAGGTTTGTGTTGTCCACATATA-3′ (wild type specific primer) and the common forward primer in another PCR reaction to show presence of wild type allele (123 base pair PCR product). Carriers were confirmed by sequencing.

**Figure 2:**
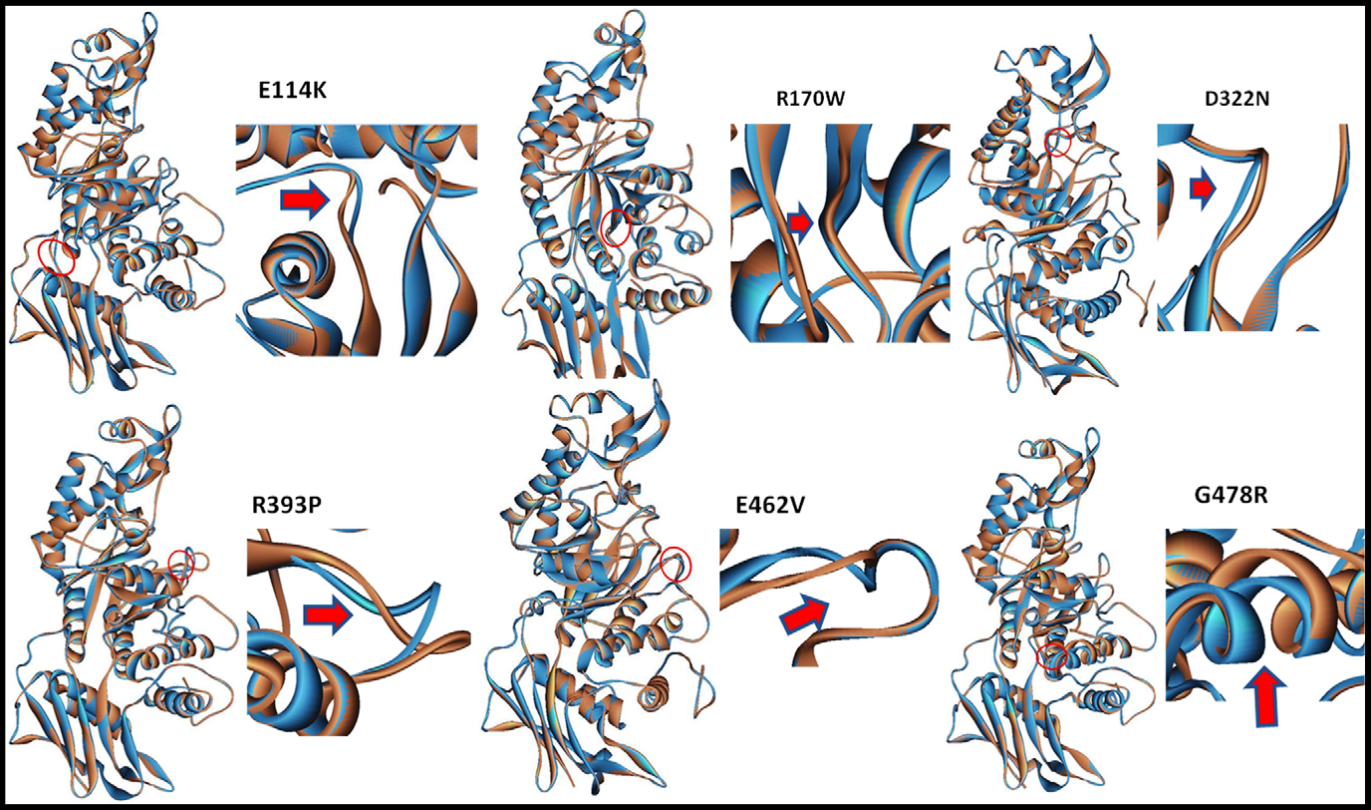
Superimposed native structures (blue) and mutant structure (brown) of the α subunit produced using Accelrys Discovery Studio software from top left clockwise: a) mutation p.E114K causes conformational change, b) p.R170W disrupts the beta sheet c) p.D322N affects the active catalytic site, d) p.R393P causes conformational change, e) p.E462V affects the active site and the dimerization of alpha-beta subunits, f) p.G478R disrupts the alpha helix.

**Figure 3:**
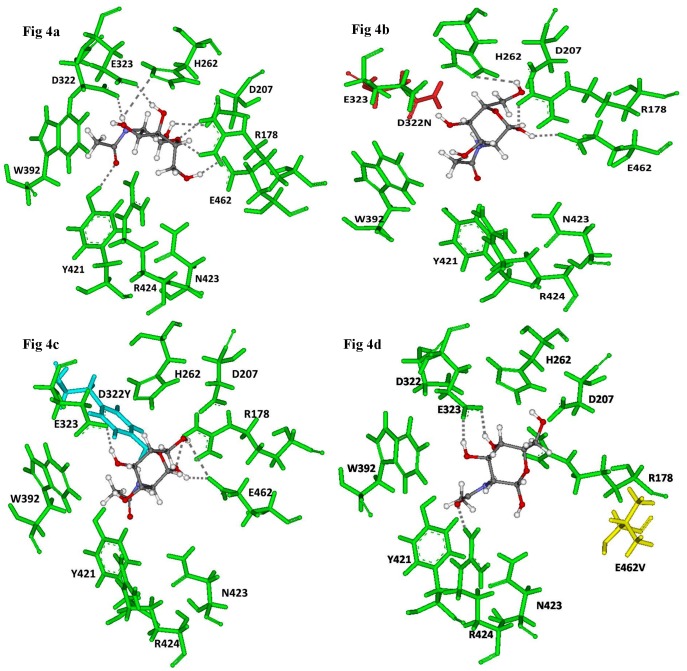
Stereoscopic view of the docking experiments. Green sticks indicate C^α^ trace of amino acids involved in the active site and CPK (Corey-Pauling-Koltun) coloring scheme represents ligand GalNAc (N acetyl galactosamine) portion of GM2 ganglioside. Fig 4a: Wild type (αHex-A-GalNAc complex). Fig 4b: Mutant (αD322N-GalNAc), Red shows mutation p.D322N, Fig 4c: Mutant (αD322Y-GalNAc), Cyan shows mutation p.D322Y, Fig. 4d: Mutant (α E462V-GalNAc), yellow shows mutation p.E462Y.

The Sorting Tolerant from Intolerant (SIFT) index (available at http://sift.jcvi.org/), Polyphen2 scores (available at http://genetics.bwh.harvard.edu/pph2/) and MutationT@ster (available at http://www.mutationtaster.org/) scores for the non-synonymous single nucleotide substitutions are given in Table 3. [7]–[9] The predictions of the SIFT, Polyphen2 and MutationT@ster algorithms were in concordance with the observed pathogenicity in case of mutations p.E114K, p.R170W, p.D322N, p.D322Y, and p.E462V. The RMSD (Root mean square deviation) values for the modeled mutants were significant for pathogenicity for all missense mutations except p.E114K (refer to Table 3 and Figure 2). This mutation was determined pathogenic by SIFT and Polyphen analysis. The docking studies reveal that the residues αAsp322, αTyr421, αArg178, αGlu462 play a crucial role in the binding and orientation of GalNAc (refer to Figure 3 and Tables 3, 4 and 5).

The intronic mutation c.672+30T>G was evaluated for pathogenicity using MutationT@ster. The mutation was predicted to lead to gain of aberrant donor splice site at position c.672+35 (score 0.5). However no functional studies could be carried out further since the family was not available later for further testing. It was not found in 100 controls with normal Hex A values. Another intronic mutation c.805+1 G>C disrupts the normal donor splice site of the intron 7. This was found in a consanguineous carrier couple from Andhra Pradesh with history of a previous child being affected with Tay Sachs disease (refer to Table 1).

**Table 3:** Details of *HEXA* missense mutations detected in infantile TSD patients and *in silico* analysis.

Exon no	Codon no.	Codon change	Amino acid change	Mutation T@ster score	SIFT Score	Polyphen2 Score (sensitivity, specificity)	RMSD between native and mutant structures	Potential Energy after minimization (Kcal/mol) (native structure energy −25289.27)	Amino acid change
2	114	GAG – AAG	Glu114Lys	3.30 (DC)	0.00 (IT)	0.998 (0.16, 0.99) (PD)	0.134 (P)	−25045.67	Acidic to basic
5	170	CGG – TGG	Arg170Trp	2.75 (DC)	0.00 (IT)	1.00 (0, 1) (PD)	0.177 (DC)	−25025.01	Basic to nonpolar
8	322	GAT – AAT	Asp322Asn	0.63 (DC)	0.00(IT)	1.00 (0,1) (PD)	0.176 (DC)	−25599.05	Acidic to Uncharged polar
8	322	GAT- TAT	Asp322Tyr	4.36 (DC)	0.00 (IT)	1.00 (0,1) (PD)	0.182 (DC)	−25476.57	Non- cyclic to cyclic
11	393	CGA - CCA	Arg393Pro	2.81 (P)	0.10(T)	0.611 (0.8, 0.82) (PD)	0.387 (DC)	−24852.34	Basic to nonpolar
12	462	GAA - GTA	Glu462Val	3.3 (DC)	0.00 (IT)	1.00 (0,1) (PD)	0.178 (DC)	−25043.90	Acidic to nonpolar
13	478	GGG- AGG	Gly478Arg	3.41 (DC)	0.06 (T)	0.878 (0.69, 0.89) (PD)	0.453 (DC)	−24950.66	Nonpolar to Basic

DC  =  Disease causing, P  =  Polymorphism, IT  =  Intolerant, T  =  Tolerant, PD  =  Probably damaging RMSD  =  root mean square deviation.

The MutationT@sterscore is taken from an amino acid substitution matrix (Grantham Matrix [3]) which takes into account the physico-chemical characteristics of amino acids and scores substitutions according to the degree of difference between the original and the new amino acid. Scores may range from 0.0 to 6.0.

The SIFT score is the normalized probability that the amino acid change is tolerated and ranges from 0 to 1. The amino acid substitution is predicted damaging is the score is < = 0.05, and tolerated if the score is >0.05.

The Polyphen2 score is the Naïve Bayes posterior probability that this mutation is damaging and thus ranges from 0 to 1.

**Table 4:** Hydrogen bond interaction for the wild type and Mutant complexes.

Complex	Protein (Residue:Atom)	Ligand Atom
**Wild Type-GalNAc**	ARG178:HH12	O2
	ARG178:HH22	O2
	TYR421:HH	O6
	TRP460:HE1	O6
	ASP207:OD1	H26
	GLU323:OE1	H28
	GLU462:OE2	H29
	HIS262:NE2	H30
	ASP322:OD2	H20
**αD322N-GalNAc**	ARG178:HH22	O1
	GLU462:OE2	H26
	HIS262:NE2	H29
**αD322Y-GalNAc**	ARG178:HH22	O1
	GLU462:OE2	H26
	GLU323:OE2	H28
	GLU462:OE2	H29
**αE462V-GalNAc**	ARG424:HH21	O6
	GLU323:OE2	H27
	GLU323:OE1	H28

**Table 5:** Ligand Binding Energy Details for wildtype and mutant α subunits.

Complex	Binding Energy (kcal/mol)	Complex
Wild Type-GalNAc	−115.94	Wild Type-GalNAc
αD322N-GalNAc	−38.87	αD322N-GalNAc
αD322Y-GalNAc	−52.22	αD322Y-GalNAc
αE462V-GalNAc	−80.88	αE462V-GalNAc

## Discussion

The clinical and neuroimaging features of infantile Tay Sachs disease seen in our patients were consistent with the defined phenotype. The results of enzyme activity measurements (Hex A expressed as percentage of total Hex activity) varied from 0% to 1.29%. This is consistent with previous observations that infantile TSD patients have values ranging from 0 to 2% [10]. Recent studies in patients from India have revealed similar values in infantile TSD patients [11]. Our study indicates that the known deleterious common mutations c.1278insTATC, c.1421+1 G>C, c.805 G>A (p.G269S), 7.6 kb deletion and the two pseudodeficiency mutations c.739C>T (R247W) and c.745C>T (R249W) are not common in Indian TSD patients.

We found three novel deleterious mutations (p.D322N, p.D322Y, p.E462V) occurring at the functionally active site of the alpha subunit of hexosaminidase A. The mutation p.R393X has been previously identified in infantile TSD patients from Persia, Turkey and Iraq populations [12]–[14]. The mutation p.E114K is proximal to the N-glycosylation site α-N115.

**Figure 4:**
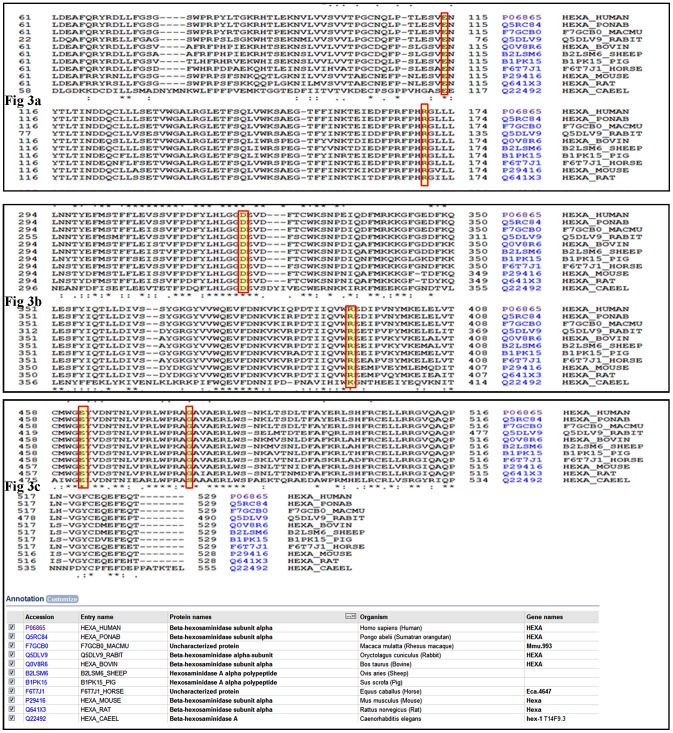
Multiple protein sequence alignment using ClustalW shows evolutionary conservation of amino acid residues. Fig. 3a: αE114 and αR170; Fig. 3b: αD322 and αR393; Fig. 3c: αE462 and αG478.

We were especially interested in analyzing the c.1385 T>A (p.E462V) mutation because it was identified in six unrelated families from one ethnic group but, to our knowledge, has never been identified in TSD patients of other ethnic origin. The sequence alignment of Hex A from various species (refer to Figure 4) reveal that the residue E462 is highly conserved. The RMSD of the modeled mutant is also significantly higher as compared to the wild-type protein (refer to Table 1 and Figure 2). This mutation was present in homozygous state in all six patients exhibiting infantile TSD. This is in accordance with previous observations that missense mutations responsible for infantile TSD were generally located in a functionally importance region, such as the active site and the dimer interface [15].

The mutation c.508C>T (p.R170W) has been previously reported in different ethnic groups [Japanese, French-Canadian (Estree region, Quebec) and Italian patients] [16]–[18]. The side chain of R170 in domain II forms a hydrogen bond with the E141 in domain I. The substitution of R with W with a bulkier side chain has a large effect on the interface of domains I and II. This would destabilize the domain interface and cause degradation of the α-subunit [16], [19].

The intronic mutation c.672+30T>G has been previously reported in an obligate carrier individual detected on biochemical screening. The mutation has been reported to be benign in this paper as it was found in combination with another disease causing mutation in an obligate carrier [20]. The National Center for Biotechnology Information (NCBI) single nucleotide polymorphism database (dbSNP) (http://www.ncbi.nlm.nih.gov/SNP/index.html) revealed that the minor allele count of this polymorphism (rs117160567) is minimal (C = 0.0103/13). However this allele has not been reported in homozygous state till date. Thus, the pathogenic potential of this mutation remains to be explored by functional analysis. The presence of another *HEXA* mutation in this patient in form of large heterozygous deletion or mutation in regions not analyzed like deep intronic and promoter cannot be ruled out.

The splice site mutation c.805+1 G>C has been reported previously from Portuguese patients [21]. Another substitution at the same nucleotide c.805+1 G>A is common in French-Canadian patients from the Quebec region in Canada [22]. Both mutations are known to be associated with the infantile form of TSD.

In one of our patients, no mutation could be identified. Complete gene sequencing strategy is likely to miss large heterozygous deletions or gene rearrangements. Southern blot analysis or multiplex ligase dependent probe amplification (MLPA) based methods to detect these changes were not available to us.

A large number of gene mutations causing TSD have been previously reported in the *HEXA* mutation database http://www.hexdb.mcgill.ca/. Among them, mutations resulting in gross alterations in the Hex α-subunit sequence are generally found in the severe infantile form. However, missense mutations causing amino acid substitutions have been found in both the infantile and late onset phenotypes [23]. Mahuran et al, (1999) reported that the detrimental effect of most mutations is on overall folding and/or transport of the protein rather than on the functional sites [24]. Some of the mutations identified in our series affect crucial active residues or cause significant structural aberrations leading to the infantile form of the disease.

### Conclusion

The mutations responsible for Tay Sachs disease in Indian population are unique. Patients originating from Gujarat state could be screened for the founder mutation p.E462V. Except for this mutation, the rest of the mutations were non-recurrent. However, screening for the above mutations would be recommended for cost effective testing of TSD patients.

## Methods

### Ethics Statement

The study protocol was approved by Ethics Committee of Foundation for Research in Genetics and Endocrinology, Ahmedabad, Gujarat (Reg No- E/13237).

### Study Subjects

Clinical details were noted in a case record form and an informed written consent was obtained from each family. The clinical criteria for inclusion in this study was history of neuroregression, exaggerated startle reflex, cherry red spots on fundus examination and relative macrocephaly. Neuroimaging criteria included presence of gray matter disease shown by hyperintensity in basal ganglia and/or hypointensity in thalamus in T2 weighted images of MRI of the brain. Five ml peripheral blood was collected from the patients for leukocyte enzyme assay and DNA extraction. Leukocyte hexosaminidase A activity was determined by manual fluorimetric enzyme assay. Total hexosaminidase (Hex) was measured from the hydrolysis of the synthetic substrate 4-methylumbelliferyl-N-acetyl-β-D-glucosamine (MUG), which releases fluorescent 4-methylumbelliferone when acted upon by β-hexosaminidase. Hexosaminidase B (Hex B) was determined as the activity left after samples were heated for three hours at 50°C. This procedure led to loss of Hex A which is heat-labile but not of hexosaminidase B (Hex B) or intermediate isoenzyme. Therefore, Hex A activity was obtained after subtracting Hex B activity from total Hex activity. Hex A was also assayed with the more specific sulphated substrate 4-methylumbelliferyl-N-acetyl-β-D-glucosamine-6-sulphate (MUGS) [25]. Hex A activity (obtained from MUGS lysis) was expressed as percentage of total Hex activity (obtained from MUG lysis).

### Screening Procedure for Common Mutations

Genomic DNA was extracted by the standard salting out method. Primary screening of the common mutations c.1277_1278insTATC, c.1421+1 G>C, 7.6 kb deletion, the pseudo-deficiency alleles (p.R247W and p.R249W) was performed by amplification refractory mutation system – polymerase chain reaction (ARMS PCR) using normal and mutant primers as a reverse primers and common forward primers as previously described [26], [27]. PCR amplification was performed for each mutation separately in a 15 ul final reaction volume containing 500 ng genomic DNA, 10× Cetus buffer, 2.0 mM dNTPs, 1 U Taq polymerase, 4–6 pmol of each primers and 1.5 µl 10× Cetus buffer. The thermocycling conditions were 10 min at 94°C, followed by 25–32 cycles of amplification consisting 30 s–60 s at 94°C, 30 s–60 s at 55–63°C and 30 s –3 min at 72°C, and a final elongation for 10 min at 72°C and PCR product were run on 2% agarose and visualized under ultraviolet transilluminator.

### Molecular Analysis of *HEXA* Gene

The exonic and intronic flanking sequence of the *HEXA* gene were PCR amplified in 14 fragments using previously described primers [28]. DNA amplification was performed for each fragment in a 10 µl final volume containing 100 ng genomic DNA, 1 mM dNTPs, 10 pmol of each primer, 1 U Taq polymerase and 1 µl 10 X PCR buffer. Thirty cycles of amplification were used, each consisting 1 min denaturation at 94°C, 45 s annealing at 60–65°C suitable for each exons and 45 s extension at 72°C in a thermal cycler. Final extension time was 10 minutes. Negative control PCR tubes contained all of the above ingredients except DNA. PCR products were then electrophoresed in 2% agarose along with the appropriate negative controls and a 100 base-pair DNA ladder. Products that passed this quality check were purified by treatment with Exo-SAP-IT ™ (USB Corporation, OH, USA) and then sequenced using BigDye Terminator v3.1 and capillary electrophoresis was performed using an automated sequencer ABI-3130 (Applied Biosystems, CA, USA). Mutations were described according to mutation nomenclature, considering nucleotide +1 the A of the first ATG translation intitiation codon. Nucleotide numbers are derived from cDNA HEXA sequence (RefSeq cDNA NM_000520.4). Putative mutations were confirmed in two separate PCR products from the patient’s DNA. Heterozygosity for these mutations was confirmed in the parents. The mutations identified were then looked up in public databases like The Human Gene Mutation Database (http://www.hgmd.cf.ac.uk), dbSNP (http://www.ncbi.nlm.nih.gov/SNP/index.html) and McGill University database (http://www.hexdb.mcgill.ca). Novel variants were also ruled out as polymorphism by sequencing the corresponding exons/introns in 100 normal unrelated individuals.

### In *Silico* Analysis

Prediction of functional effects of non-synonymous single nucleotide substitutions (nsSNPs) was done using softwares SIFT (Sorting Intolerant From Tolerant) (available at http://sift.jcvi.org/), Polyphen2 (Polymorphism Phenotyping v2) (available at http://genetics.bwh.harvard.edu/pph2/) and MutationT@ster (available at http://www.mutationtaster.org/). [7]–[9] HumVar-trained prediction model of Polyphen2 was used for distinguishing mutations with drastic effects from all the remaining human variation, including abundant mildly deleterious alleles. Evolutionary conservation of the amino acid residues of Hex A was analyzed using ClustalW program available online at http://www.uniprot.org/help/sequence-alignments.

### Structural Studies

#### Protein Preparation

The mutants (αD322N, αD322Y, αE462V )were built using build mutant protocol of Accelrys Discovery studio 2.0 using the crystallographic structure of Hex A (PDB ID: 2 GJX ) as the template. The wild type protein and mutants were energy minimized using CHARMm forcefield and 200 cycles of steepest descent algorithm.

#### Ligand Preparation

The structural coordinates of GM2 ganglioside was retrieved from the PubChem database (CID 9898635). The GalNAc (N-acetyl galactosamine) portion of the GM2 ganglioside was considered for docking on the active site of Hex A as reported previously [29]. It was prepared using the Prepare Ligands protocol of Accelrys Discovery studio 2.0.

#### Molecular Docking

Structural analysis revealed that residues R178, D207, H262, E323, D322, W373, W392, Y421, N423, R424 and E462 constitute the active site of αHex-A. This information was used to define the binding site and docking studies were performed using the LigandFit protocol of Accelrys Discovery studio 2.0. The potential energy and Root Mean Square Deviations (RMSD) of the mutant structures with respect to the wild-type structure were calculated. RMSD values more than 0.15 were considered as significant structural perturbations that could have functional implications for the protein [15].

## Acknowledgments

We would like to thank the Director of NIRRH, Dr. Sanjiva Kholkute for his support. We also thank Rochelle Tixeira for technical assistance. We thank the families of patients with Tay Sachs disease for their participation in this research.
